# Diosmetin Ameliorates Vascular Dysfunction and Remodeling by Modulation of Nrf2/HO-1 and p-JNK/p-NF-κB Expression in Hypertensive Rats

**DOI:** 10.3390/antiox10091487

**Published:** 2021-09-17

**Authors:** Sariya Meephat, Patoomporn Prasatthong, Prapassorn Potue, Sarawoot Bunbupha, Poungrat Pakdeechote, Putcharawipa Maneesai

**Affiliations:** 1 Department of Physiology, Faculty of Medicine, Khon Kaen University, Khon Kaen 40002, Thailand; sariya_m@kkumail.com (S.M.); pa_pra@kkumail.com (P.P.); prappo@kku.ac.th (P.P.); ppoung@kku.ac.th (P.P.); 2 Faculty of Medicine, Mahasarakham University, Mahasarakham 44000, Thailand; sarawoot.b@msu.ac.th; 3 Research Institute for Human High Performance and Health Promotion, Khon Kaen University, Khon Kaen 40002, Thailand

**Keywords:** diosmetin, vascular function, vascular remodeling, oxidative stress, inflammation

## Abstract

Diosmetin is a citrus flavonoid that has antioxidant and anti-inflammatory effects. This study examined the effect of diosmetin on blood pressure and vascular alterations and its underlying mechanisms in experimentally hypertensive rats. Male Sprague rats were administered Nω-nitro-l-arginine methyl ester L-NAME for five weeks and were given diosmetin at doses of 20 or 40 mg/kg or captopril (5 mg/kg) for two weeks. Diosmetin alleviated hypertension, improved endothelial dysfunction, and suppressed the overactivity of sympathetic nerve-mediated vasoconstriction in aorta and mesentery hypertensive rats (*p* < 0.05). Increases in plasma and aortic tissue malondialdehyde (MDA) and carotid superoxide generations and reductions of plasma superoxide dismutase, catalase, and nitric oxide in hypertensive rats were ameliorated by diosmetin (*p* < 0.05). Diosmetin increased the protein expression of nuclear factor erythroid 2-related factor 2 (Nrf2) and heme oxygenase-1 (HO-1) in hypertensive rats. Furthermore, diosmetin mitigated hypertrophy and collagen accumulation of the aortic wall in L-NAME rats. It exhibited an anti-inflammatory effect by reducing interleukin-6 (IL-6) accumulation and by overexpressing the phospho-c-Jun N-terminal kinases (p-JNK) and the phospho-nuclear factor-kappaB (p-NF-κB) proteins in the aorta (*p* < 0.05). Captopril was a positive control substance and had similar effects to diosmetin. In summary, diosmetin reduced blood pressure and alleviated vascular abnormalities in L-NAME-treated rats. These effects might be related to antioxidant and anti-inflammatory effects as well as to the modulation of the expression of the Nrf2/HO1 and p-JNK/NF-κB proteins.

## 1. Introduction

High blood pressure is a chronic disease and a main cause of cardiovascular disease as well as vital organ damage. An imbalance of the vasoactive agents derived from the vascular endothelium and the overactivity of sympathetic nerve-innervated vasculature are well documented in the development and maintenance of hypertension [1]. Nitric oxide (NO) is an essential vasodilator released from the vascular endothelium in response to acetylcholine to reduce vascular tone [2]. Depletion of NO by Nω-nitro-l-arginine methyl ester (L-NAME), a non-specific nitric oxide synthase (NOS) inhibitor, has been established as a treatment to induce [3]. It has been reported that long-term treatment with L-NAME causes sustained hypertension, cardiovascular dysfunction, and remodeling in animals [4,5,6,7]. Moreover, sympathetic nerve-mediated contractile responses are enhanced in the vasculature isolated from NOS inhibitor-treated rats, which is relevant to a high level of plasma norepinephrine and a low level of plasma NO in these animal models [8,9,10]. Vascular structural changes such as vascular hypertrophy and fibrosis have been observed in NO-deficient rats [5,11]. These changes are associated with adaptive responses to a high-pressure load [6], and the underlying mechanisms are possibly mediated by oxidative stress and inflammation.

Oxidative stress is caused by the overproduction of reactive oxygen species (ROS) together with a low level of endogenous antioxidant enzyme activity or production [12]. It has been suggested that oxidative stress is one of the underlying mechanisms that maintains hypertension in L-NAME-treated rats. This is supported by evidence that endothelial dysfunction in L-NAME rats mainly results from decreased NO bioavailability and oxidative stress [13,14]. ROS, such as superoxide (O_2_^•−^), can suppress NO bioavailability by reacting with NO to produce peroxynitrite (ONOO^−^) [15]. However, after exposure to oxidative stress, the cellular defense mechanisms are initiated by increasing the activity of the erythroid 2-related factor 2 (Nrf2), a transcriptional factor [16]. Under excessive ROS production, Nrf2 subsequently translocates into the nucleus and transcripts antioxidant enzymes, such as heme oxygenase-1 (HO-1), superoxide dismutase (SOD), and catalase (CAT), against ROS [17,18]. The activation of the Nrf2 signaling pathway can alleviate oxidative stress, organ damage, and inflammation in methotrexate-treated rats [19]. In L-NAME hypertensive rats, reductions in Nrf2/HO-1 expression associated with low levels of endogenous antioxidant enzymes have been reported [5]. Additionally, excessive ROS production can induce inflammation via the activation of nuclear factor-kappaB (NF-κB). This is supported by evidence that ROS can activate the mitogen-activated protein kinase (MAPK) component and NF-κB pathways to mediate cell growth, proliferation, and inflammation [20]. Kalra and co-workers reported that the administration of L-NAME increases the expression of phosphor c-Jun N-terminal kinases (p-JNK), while NF-κB protein expression leads to tissue inflammation [21].

Diosmetin is the aglycone of the flavonoid glycoside diosmin, which is mostly found in citrus fruits such as orange, lemons, and grapefruit [22]. Diosmetin is isolated from seeds of *Acacia farnesiana* and Portuguese olive (*Olea europaea* L.) leaves [23,24]. Over more than three decades, a wide range of therapeutic effects for diosmetin have been demonstrated. Diosmetin exhibits an excellent antioxidant capacity, both in vivo and in vitro [25,26]. The anti-inflammatory effects of diosmetin have been observed in dinitrochlorobenzene-induced atopic dermatitis in mice [27]. In streptozotocin-induced diabetic nephropathy mice, treatment with diosmetin can alleviate the signs of diabetes, which are associated with reducing oxidative stress and inflammatory markers in the serum and tissue [28]. Furthermore, diosmetin prevents lipopolysaccharide-induced acute lung injury in mice via the activation of Nrf2/HO-1 expression and the suppression of inflammation [29]. A previous study found that diosmetin alleviates the signs of cardiometabolic disorders and improves left ventricular dysfunction and remodeling in high-fat diet fed rats [30]. Recently, the direct effects of diosmetin on the vascular response were tested, as it produces a concentration-dependent relaxation of the porcine coronary artery under raised tone conditions with U46619 [31]. Captopril is an angiotensin-converting enzyme inhibitor (ACEI) that is widely used for hypertension management. Other potential effects of captopril have also been reported, including antioxidation, anti-inflammation, cardio-protection, and vasodilation [32,33,34]. In this study, captopril was used as a positive control agent. The aim of this study was to explore the beneficial effects of diosmetin on hypertension, vascular dysfunction, and remodeling as well as the mechanism involved in hypertensive rats.

## 2. Materials and Methods

### 2.1. Chemical and Antibodies

Diosmetin was bought from ChemFaces Bioschemical Co., Ltd. (Wuhan, China). L-NAME and captopril were supplied by Sigma-Aldrich Corp. (St Louis, MO, USA). This study primarily used mouse antibodies to Nrf2, HO-1, IL-6 (Santa Cruz Biotechnology, Inc., Santa Cruz, CA, USA), and p-JNK and the rabbit primary antibody to p-NF-κB (Cell Signaling Technology, Inc., Danvers, MA, USA). A second antibody, goat anti-mouse antibody (Abcam Plc, Cambridge, UK), was used in this study. The intensity of β-actin protein expression was used as a control protein.

### 2.2. Animal and Protocols

In the present study, male Sprague Dawley rats weighing 220–240 g were obtained from Nomura Siam International Co, Ltd., Bangkok, Thailand. For an adaptation period, all of the rats were housed in a controlled environment before the experiment. Thereafter, the rats were allocated into five groups (*n* = 8/group) comprising a control group (received propylene glycol as the vehicle), an LN group (received L-NAME in drinking water at a dose of 40 mg/kg for 5 weeks), an LN + Diosmetin20 group (LN treated with diosmetin at a dose of 20 mg/kg), an LN + Diosmetin40 group (LN treated with diosmetin at a dose of 40 mg/kg), and an LN + Captopril (LN treated with captopril at a dose of 5 mg/kg). The rats were treated with either diosmetin or captopril for the final two weeks. All procedures were permitted by the Animal Ethics Committee of Khon Kaen University, Khon Kaen, Thailand (Permit No. IACUC-KKU-70/61). The choice of L-NAME, diosmetin, and captopril doses was influenced by previous studies [5,30].

### 2.3. Blood Pressure Measurement in Conscious and Anesthetized Rats

Indirect blood pressure measurements were performed once a week in all of the rats using tail-cuff plethysmography (IITC/Life Science Instrument, Woodland Hills, CA, USA). Three repeated values of systolic blood pressure (SP) were taken, and the data are presented as the mean ± SEM. The direct method of blood pressure measurement was performed under anesthetized conditions (intraperitoneal injection of thiopental sodium (70 mg/kg)) at the end of the study. The femoral artery was cannulated, and the values of SP, diastolic blood pressure (DP), mean arterial pressure (MAP), and heart rate (HR) were then collected using Acknowledge Data Acquisition software (Biopac Systems Inc., Santa Barbara, CA, USA). These data are presented as the mean ± SEM. 

### 2.4. Vascular Function Study

The mesenteric vascular beds were removed and set on a stainless-steel grid in a humid controlled temperature chamber. The preparations were perfused with 37 °C oxygenated physiological Krebs’ solution for 30 min to achieve a stable baseline. Subsequently, capsaicin (0.1 µM) was perfused to abolish the effect of sensory nerve stimulation followed by a 30 min wash out period by perfusion with Krebs’ solution. To see the sympathetic nerve-mediated contractile function of the mesenteric vascular beds, electrical filed stimulation (EFS) was conducted at 5–40 Hz, 90 V, and 1 ms for 30 s at 5 min intervals followed by a bolus injection of norepinephrine (NE) (0.15–15 nmol). To assess the vasorelaxation responses to various vasoactive agents, a bolus injection of acetylcholine (ACh) (at 10^–8^ to 10^–4^ M) or sodium nitroprusside (SNP) (10^–8^ to 10^–4^ M) was applied under raised tone (90–110 mmHg above baseline) conditions. Changes in the vascular response were noticed by a pressure transducer and were then amplified by the BIOPAC System (BIOPAC System Inc., Aero Camino Goleta, CA, USA). The thoracic aorta was carefully removed, cleaned, and cut into rings. The rings were hung in 15 mL organ baths containing 37 °C physiological Krebs’ solution with a resting tension of 1 g. After raising the tone with 10 µM phenylephrine, ACh (0.01–3 µM) or SNP (0.01–3 µM) was cumulatively added into the bath to assess the endothelium-dependent and endothelium-independent vasorelaxation.

### 2.5. Oxidative Stress Marker Assessments

#### 2.5.1. Superoxide (O_2_^•−^) Production and Malondialdehyde (MDA) Levels

The lucigenin-enhanced chemiluminescence principal method [33] was used to determine the production of vascular O_2_^•−^ production in the carotid artery. The level of thiobarbituric acid reactive substances (TBARs) expressed in the MDA equivalent was assessed in the plasma and aortic tissue based on a previously described method [35].

#### 2.5.2. Plasma Catalase (CAT) and Superoxide Dismutase (SOD) Activities

CAT activity was measured in plasma, as previous described by Goth in 1991 and Ozmen et al. in 2002 [36,37]. Briefly, the plasma samples were mixed with 30% H_2_O_2_ and were then incubated for 1 min at 37 °C. Thereafter, 100 µL of ammonium molybdate was added to the mixture to stop the enzymatic reaction. The absorbance values were achieved by using a spectrophotometric detector at 405 nm, and the data are expressed as units per milliliter.

The activity of the plasma SOD was determined by a commercial ELISA Kit (ELISA kit-19160, Sigma-Aldrich, Darmstadt, Germany) following the manufacturer’s instructions.

#### 2.5.3. Plasma Nitrate/Nitrite (NOx) Level

The level of NOx was measured in the plasma by the conversion of nitrate to nitrite using nitrate reductase, followed by Griess reagents, as previously published [38].

### 2.6. Histological and Immunohistochemical Studies

The aortic tissue was fixed in 4% formalin before being dehydrated, embedded, and cut into 5 µm-thick sections. Vascular morphometric changes in the thoracic aorta were evaluated using the hematoxylin and eosin (H&E) and picrosirius red staining techniques. Cross-sectional area, thoracic wall thickness, lumen diameter, wall/lumen ratio, and vascular smooth muscle cells were observed under a light microscope (Nikon, Tokyo, Japan) and the percentage of vascular fibrosis was observed under polarized light microscope (Nikon, Tokyo, Japan). The expression of IL-6 was also evaluated in the aorta using immunohistochemistry staining by means of the incubation of primary antibody IL-6 (Catalog Number sc57315, dilution 1:500) for 4 h in a moistening chamber under room temperature and followed by incubation with secondary antibodies for 2 h at room temperature. The level of IL-6 was expressed as the percentage of the relative strained areas, as per a previous report [39].

### 2.7. Western Blot Analysis

Nrf-2 (Catalog Number sc365949), HO-1 (Catalog Number sc136960), p-JNK (Catalog Number 9255S), and p-NF-κB (Catalog Number 3033S) protein expression in the thoracic aorta were measured using the Western blot method following a previously published method (Bunbupha et al., 2014). The thoracic aorta was homogenized, and the proteins were electrophoresed on a sodium dodecylsulfate polyacrylamide gel electrophoresis system. Thereafter, the proteins were electrotransfered onto a polyvinylidenedifluoride membrane and were blocked with 5% skimmed milk in Tris-buffered saline (TBS) with 0.1% Tween 20 for 2 h at room temperature before overnight incubation at 4 °C with mouse monoclonal antibodies to Nrf-2, HO-1, p-JNK; rabbit polyclonal antibodies to p-NF-kB; and goat polyclonal IgG to β-actin. After the incubation period, the membranes were washed with TBS and were then incubated for 2 h at room temperature with horseradish peroxidase conjugated secondary antibody. The blots were developed in LuminataTM Forte Western HRP Substrate (Millipore Corp., Billerica, MA, USA), and densitometric analysis was performed using an Amersham Imager 600 (GE Healthcare Life Sciences, Uppsala, Sweden). The intensity of the tbands was normalized to that of β-actin, and data were expressed as a percentage of the values determined in control group from the same gel.

### 2.8. Statistical Analysis

The data were analyzed for significance using GraphPad prism (version 8.3). A one-way analysis of variance (ANOVA) followed by Tukey’s post hoc test was used for statistical analysis. A *p*-value of <0.05 was considered statistically significant.

## 3. Results

### 3.1. Effect of Diosmetin and Captopril on Blood Pressure

**Figure 1:**
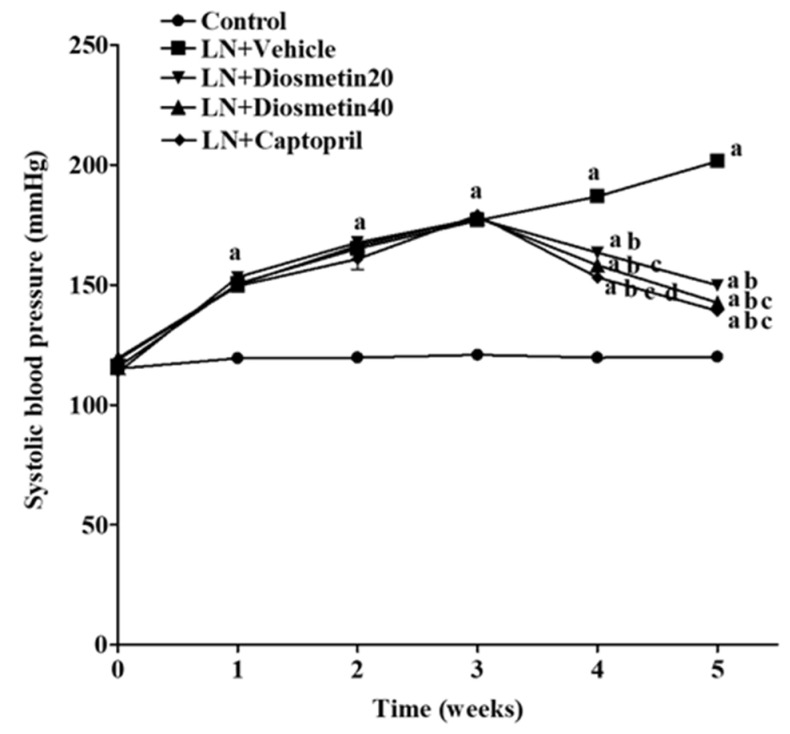
Changes in systolic blood pressure in conscious rats throughout the experimental period. ^a^ *p* < 0.05 vs. control group, ^b^ *p* < 0.05 vs. LN + vehicle group, ^c^ *p* < 0.05 vs. LN + diosmetin20 group, ^d^ *p* < 0.05 vs. LN + diosmetin40 group. LN, L-NAME; LN + diosmetin20, L-NAME treated with 20 mg/kg of diosmetin; LN + diosmetin40, L-NAME treated with 40 mg/kg of diosmetin; LN + captopril, L-NAME treated with captopril (*n* = 8/group). Statistical analysis between the five groups was performed using the one-way ANOVA followed by Turkey’s post hoc test.

The effect of diosmetin and captopril supplementation on systolic blood pressure (SP) is shown in Figure 1. At the beginning of the experiment, the SP values in all of the rats were similar. The L-NAME-treated rats showed a gradual increase in SP throughout the five weeks of the experimental period (SP at week 5, 201.50 ± 2.48 mmHg) compared to those of the control rats (SP at week 5, 119.96 ± 1.64 mmHg) (*p* < 0.05). Diosmetin supplementation at doses of 20 and 40 mg/kg significantly decreased SP compared to untreated rats (SP at week 5, 149.83 ± 1.42 and 143.38 ± 0.61 mmHg) (*p* < 0.05). Moreover, rats treated with captopril (5 mg/kg) showed a significant decrease in SP (SP at week 5, 138.67 ± 0.75 mmHg) when compared to the LN and diosmetin treated groups (*p* < 0.05) (Figure 1). Similar results for SP were found in the hemodynamic parameters, as shown in Table 1. The LN groups that received diosmetin exhibited reductions in all hemodynamic parameters in comparison to the untreated rats (*p* < 0.05). In addition, captopril produced a greater effect in terms of reducing SP and MAP compared to the diosmetin20 group (*p* < 0.05) (Table 1). However, there were no significant differences in any of the hemodynamic parameters between the captopril and diosmetin groups at a dose of 40 mg/kg.

### 3.2. Effect of Diosmetin and Captopril on Vascular Function

#### 3.2.1. Effect of Diosmetin and Captopril on the Contractile Response to EFS and Exogenous NE in the Mesenteric Vascular Bed

**Figure 2:**
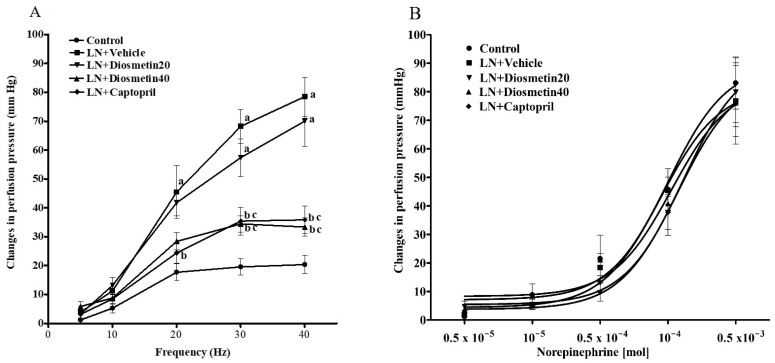
Effect of diosmetin and captopril on the contractile response to EFS (**A**) and exogenous NE (**B**) in the mesenteric vascular bed. ^a^ *p* < 0.05 vs. control group, ^b^ *p* < 0.05 vs. LN + vehicle group, ^c^ *p* < 0.05 vs. LN + diosmetin20 group. LN, L-NAME; LN + diosmetin20, L-NAME treated with 20 mg/kg of diosmetin; LN + diosmetin40, L-NAME treated with 40 mg/kg of diosmetin; LN + captopril, L-NAME treated with captopril (*n* = 8/group). Statistical analysis between the five groups was performed using the one-way ANOVA followed by Turkey’s post hoc test.

A frequency-dependent contractile response to EFS was detected in all preparations (Figure 2A). The contractile response to EFS was increased in the preparation isolated from the LN rats compared to the response in the control rats (at 40 Hz, 78.53 ± 6.74 vs. 20.38 ± 3.16 mmHg, *p* < 0.05). Interestingly, the contractile responses from the LN rats treated with diosmetin (40 mg/kg) and captopril were attenuated compared to the untreated rats (at 40 Hz, 33.38 ± 3.14 mmHg, *p* < 0.05 and 35.86 ± 4.81 mmHg, *p* < 0.05, respectively). However, the contractile response to exogenous NE (0.1 µM–0.1 mM) was similar in all preparations (Figure 2B).

#### 3.2.2. Effect of Diosmetin and Captopril on the Vascular Response to Vasoactive Agents in the Mesenteric Vascular Bed and Thoracic Aorta

**Figure 3:**
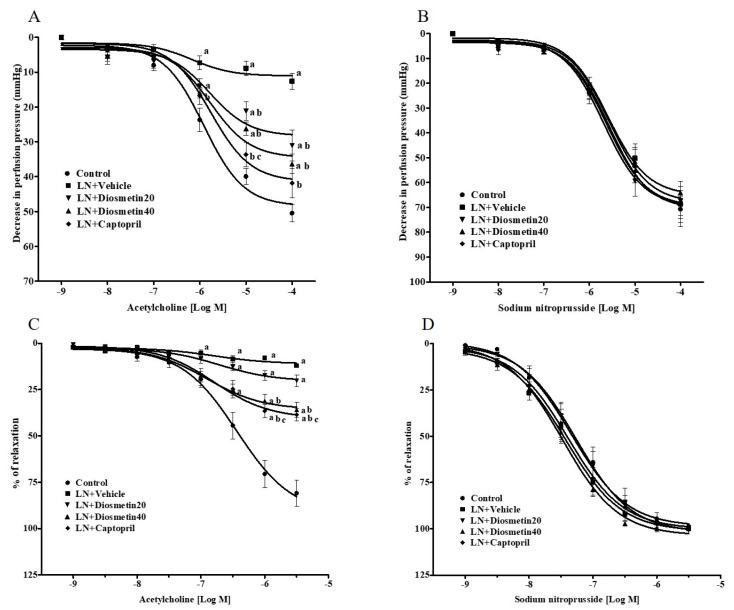
Effect of diosmetin and captopril on the vascular response to exogenous acetylcholine (**A**) and sodium nitroprusside (**B**) in the mesenteric vascular bed and exogenous acetylcholine (**C**) and sodium nitroprusside (**D**) aortic rings. ^a^ *p* < 0.05 vs. control group, ^b^ *p* < 0.05 vs. LN + vehicle group, ^c^ *p* < 0.05 vs. LN + diosmetin20 group. LN, L-NAME; LN + diosmetin20, L-NAME treated with 20 mg/kg of diosmetin; LN + diosmetin40, L-NAME treated with 40 mg/kg of diosmetin; LN + captopril, L-NAME treated with captopril (*n* = 8/group). Statistical analysis between the five groups was performed using the one-way ANOVA followed by Turkey’s post hoc test.

The vascular response to ACh in the mesenteric preparations isolated from the hypertensive rats was decreased compared to those of the control preparations (0.1 mM, 12.62 ± 2.28 vs. 50.48 ± 2.47 mmHg) (*p* < 0.05). Diosmetin and captopril improved the vasorelaxation responses to ACh compared to the untreated group (0.1 mM, D20: 31.06 ± 4.49, D40: 36.30 ± 2.75, and Cap: 41.81 ± 4.23 mmHg (*p* < 0.05)) (Figure 3A). However, the relaxation response to SNP did not differ among the groups, as shown in Figure 3B. Moreover, there was a significant decrease in the endothelium-dependent relaxation in the aortic rings from the LN group compared to the control group (3 µM, 12.18 ± 0.92% vs. 81.02 ± 7.03% of relaxation) (*p* < 0.05). Supplementation with diosmetin (40 mg/kg) and captopril improved the vasorelaxation response to ACh compared to the untreated group (3 µM, 35.96 ± 4.10% and 38.62 ± 3.17% of relaxation) (*p* < 0.05) (Figure 3C). Additionally, the response to SNP was similar in all groups (Figure 3D).

### 3.3. Effect of Diosmetin and Captopril on Oxidative Stress Markers

**Figure 4:**
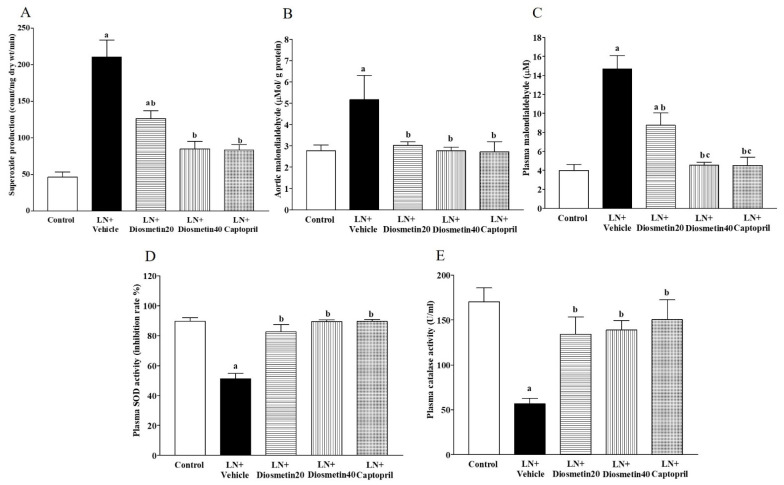
Effect of diosmetin and captopril on vascular O_2_^•−^ production (**A**), aortic MDA levels (**B**), plasma MDA levels (**C**), plasma SOD activity (**D**), and plasma CAT activity (**E**) in LN-induced hypertensive rats. ^a^ *p* < 0.05 vs. control group, ^b^ *p* < 0.05 vs. LN + vehicle group, ^c^ *p* < 0.05 vs. LN + diosmetin20 group. LN, L-NAME; LN + diosmetin20, L-NAME treated with 20 mg/kg of diosmetin; LN + diosmetin40, L-NAME treated with 40 mg/kg diosmetin; LN + captopril, L-NAME treated with captopril (*n* = 7–8/group). Statistical analysis between the five groups was performed using the one-way ANOVA followed by Turkey’s post hoc test.

There were significant increases in vascular O_2_^•−^ production and vascular and plasma MDA levels in the LN group compared to the control group (Figure 4A–C) (*p* < 0.05). These alterations were improved by the diosmetin and captopril treatments (*p* < 0.05). The plasma SOD and CAT activities were decreased in the LN group, while supplementation with diosmetin and captopril restored these activities (*p* < 0.05) in the hypertensive rats, as shown in Figure 4D,E, respectively.

### 3.4. Effect of Diosmetin and Captopril on Plasma NOx Concentration

**Figure 5:**
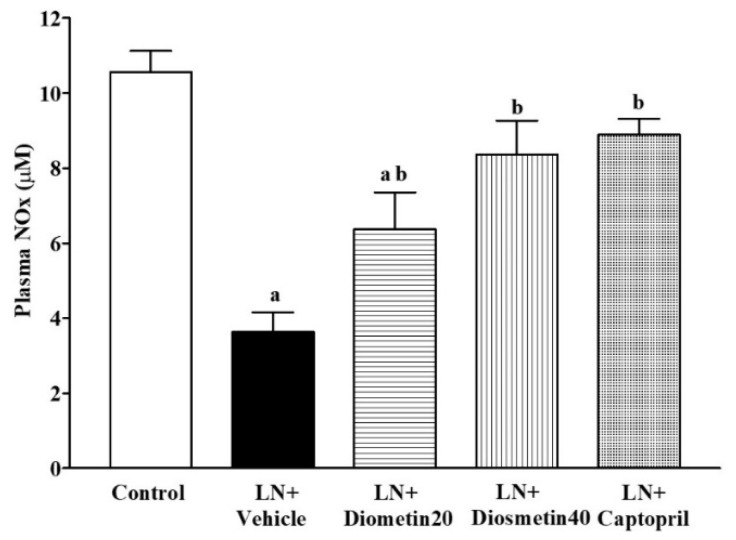
Effect of diosmetin and captopril on the plasma NOx concentration in LN-induced hypertensive rats. ^a^ *p* < 0.05 vs. control group, ^b^ *p* < 0.05 vs. LN + vehicle group. LN, L-NAME; LN + diosmetin20, L-NAME treated with 20 mg/kg of diosmetin; LN + diosmetin40, L-NAME treated with 40 mg/kg of diosmetin; LN + captopril, L-NAME treated with captopril (*n* = 7–8/group). Statistical analysis between the five groups was performed using the one-way ANOVA followed by Turkey’s post hoc test.

The plasma NOx concentrations were reduced in the LN untreated group compared to the control group (*p* < 0.05). Treatment with diosmetin and captopril ameliorated the reduction of plasma NOx induced by L-NAME (*p* < 0.05) (Figure 5).

### 3.5. Effect of Diosmetin and Captopril on Nrf2 and HO-1 Protein Expression in Vascular Tissue

**Figure 6:**
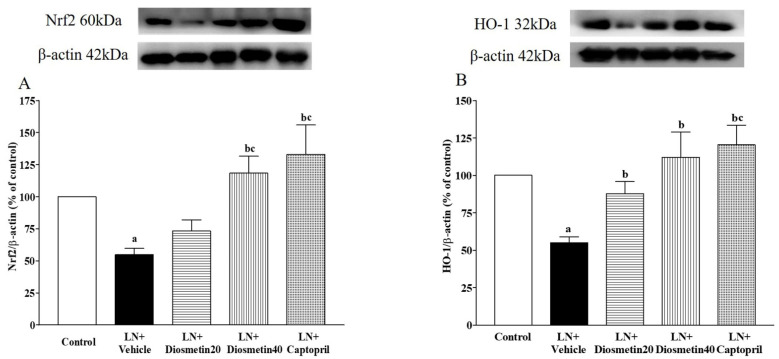
Effect of diosmetin and captopril on Nrf2 (**A**) and HO-1 (**B**) protein expression in vascular tissue. ^a^ *p* < 0.05 vs. control group, ^b^ *p* < 0.05 vs. LN + vehicle group, ^c^ *p* < 0.05 vs. LN + diosmetin20 group. LN, L-NAME; LN + diosmetin20, L-NAME treated with 20 mg/kg of diosmetin; LN + diosmetin40, L-NAME treated with 40 mg/kg of diosmetin; LN + captopril, L-NAME treated with captopril (*n* = 4/group). Statistical analysis between the five groups was performed using the one-way ANOVA followed by Turkey’s post hoc test. scale bar = 100 μm.

As shown in Figure 6A,B, the LN group had a downregulation of Nrf2 and HO-1 protein expression compared to the control group (*p* < 0.05). Diosmetin and captopril administration significantly improved the expression of Nrf2 and HO-1 protein altered by L-NAME (*p* < 0.05).

### 3.6. Effect of Diosmetin and Captopril on Vascular Morphology

**Figure 7:**
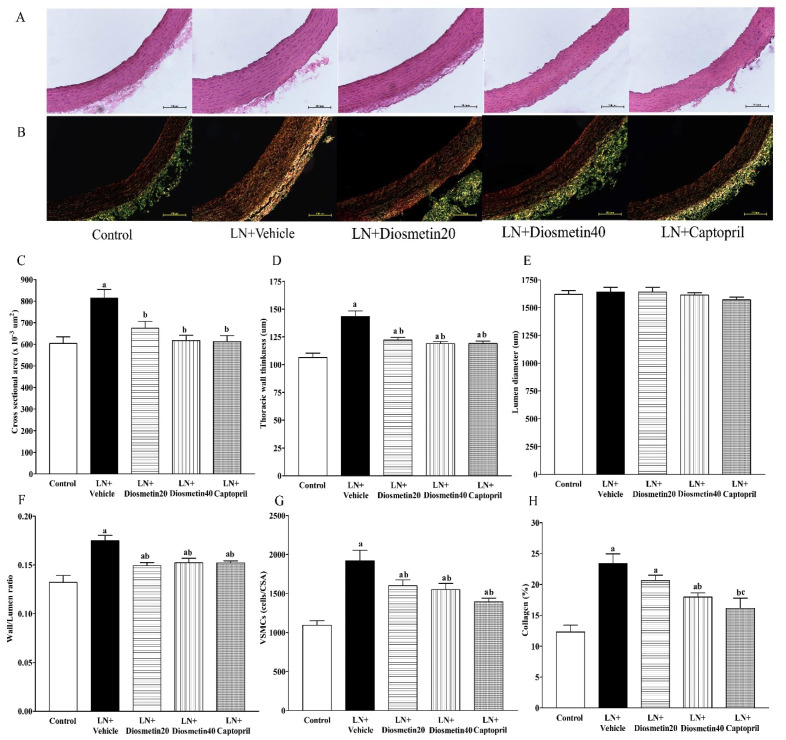
Effect of diosmetin and captopril on the vascular morphology in LN-induced hypertensive rats. Representative figures of a vascular section stained with H&E (**A**) and picrosirius red staining (**B**). Results of the quantitative analyses of vascular remodeling are represented by cross-sectional areas (**C**), thoracic wall thickness (**D**), luminal diameter (**E**), ratios of the wall to lumen (**F**), vascular smooth muscle cell numbers (**G**), and the percentage of collagen deposition (**H**). ^a^ *p* < 0.05 vs. control group, ^b^ *p* < 0.05 vs. LN + vehicle group, ^c^ *p* < 0.05 vs. LN + diosmetin20 group. LN, L-NAME; LN + diosmetin20, L-NAME treated with 20 mg/kg of diosmetin; LN + diosmetin40, L-NAME treated with 40 mg/kg of diosmetin; LN + captopril, L-NAME treated with captopril (*n* = 7–8/group). Statistical analysis between the five groups was performed using the one-way ANOVA followed by Turkey’s post hoc test.

As depicted in Figure 7A–H, the LN group showed significant increases in the cross-sectional area, vascular wall thickness, wall/lumen ratios, and vascular smooth muscle cell numbers compared to the control group (*p* < 0.05). Treatment with diosmetin and captopril considerably decreased the alteration of vascular morphology altered by L-NAME (*p* < 0.05). However, the luminal diameters did not differ between groups, as shown in Figure 7E. Interestingly, an increase in the vascular collagen content in hypertensive rats was also alleviated by diosmetin and captopril supplementation (*p* < 0.05) (Figure 7H).

### 3.7. Effect of Diosmetin and Captopril on Vascular IL-6 Expression

**Figure 8:**
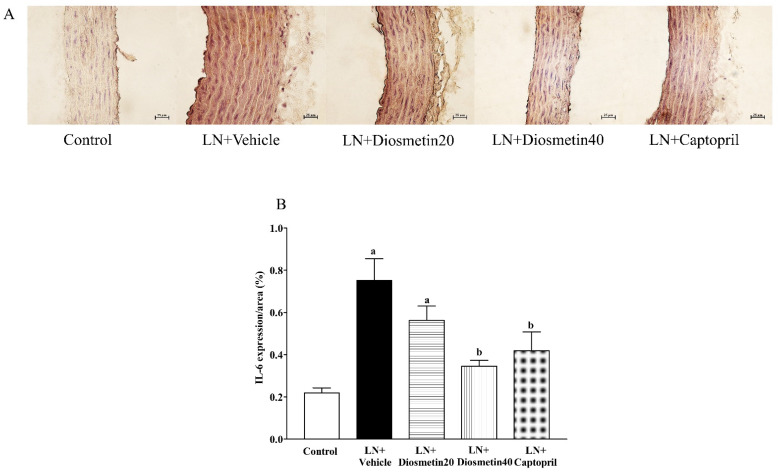
Effect of diosmetin and captopril on interleukin-6 (IL-6) immunohistochemical staining for vascular tissue (**A**) and IL-6 level (**B**) in LN-induced hypertensive rats. ^a^ *p* < 0.05 vs. control group, ^b^ *p* < 0.05 vs. LN + vehicle group. LN, L-NAME; LN + diosmetin20, L-NAME treated with 20 mg/kg of diosmetin; LN + diosmetin40, L-NAME treated with 40 mg/kg of diosmetin; LN + captopril, L-NAME treated with captopril (*n* = 7–8/group). Statistical analysis between the five groups was performed using the one-way ANOVA followed by Turkey’s post hoc test.

Immunohistochemical staining showed the enhancement of IL-6 expression in the LN group compared to the control group (*p* < 0.05). Treatment with diosmetin and captopril alleviated the expression of IL-6 (*p* < 0.05), as shown in Figure 8A,B.

### 3.8. Effect of Diosmetin and Captopril on Vascular p-JNK/p-NF-κB Protein Expression

**Figure 9:**
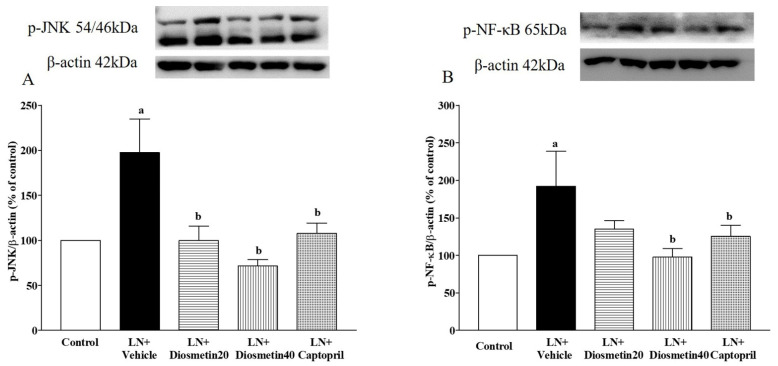
Effect of diosmetin and captopril on the p-JNK/p-NF-κB protein expression in vascular tissue (**A**,**B**) in LN-induced hypertensive rats. ^a^ *p* < 0.05 vs. control group, ^b^ *p* < 0.05 vs. LN + vehicle group. LN, L-NAME; LN + diosmetin20, L-NAME treated with 20 mg/kg of diosmetin; LN + diosmetin40, L-NAME treated with 40 mg/kg of diosmetin; LN + captopril, L-NAME treated with captopril (*n* = 4/group). Statistical analysis between the five groups was performed using the one-way ANOVA followed by Turkey’s post hoc test.

Upregulation of the p-JNK/p-NF-κB protein expression in vascular tissue was detected in the LN rats (*p* < 0.05). Administration of diosmetin and captopril decreased the overexpression of the p-JNK/p-NF-κB proteins in the L-NAME-induced hypertensive rats (*p* < 0.05) (Figure 9A,B).

## 4. Discussion

This study demonstrated that diosmetin has antihypertensive effects on L-NAME- induced high blood pressure in rats. Diosmetin also alleviates endothelial dysfunction and overactivation of sympathetic nerve-mediated vasoconstriction in conduit and small arteries in hypertensive rats. Aortic hypertrophy and fibrosis (increased collagen contents) were observed in L-NAME-treated rats, and these were mitigated by diosmetin treatment. These effects were accompanied reductions in systemic and tissue oxidative stress markers (MDA and O_2_^•−^) as well as enhancements in antioxidant enzymes (SOD and CAT), which were associated with restoration of the Nrf2/HO-1 protein expression in vascular tissue. Diosmetin also suppressed the enhancement of IL-6 staining in aortic tissue and downregulated the p-JNK/p-NF-κB protein expression in L-NAME rats. Meanwhile, captopril alleviated L-NAME-induced blood pressure elevation as well as vascular dysfunction and remodeling. It also reduced oxidative stress and inflammation and restored the Nrf2/HO-1 and p-JNK/p-NF-κB protein expressions in L-NAME-treated rats.

L-NAME causing systemic hypertension related to decreasing NO bioavailability resulting in vascular dysfunction and hypertension in animals has been noted [5,40,41]. This study found that the rats that received L-NAME had high blood pressure and decreased ACh-induced vascular relaxation in isolated aortic and mesenteric preparations. Additionally, the vasorelaxation to the NO donor, SNP, was not different between the groups. These results suggest that endothelial dysfunction occurred in this animal model. As several studies have reported, the major contribution of L-NAME-induced hypertension is vascular dysfunction [4,42]. Sympathetic nerve overactivity was observed as the contractile response to EFS was increased, but not to exogenous NE in the hypertensive group. An increased sympathetic nerve-mediated contractile response in the mesenteric vascular bed has been observed in L-NAME rats [7]. This was supported by the evidence that increased central sympathetic drive is the important mechanism contributing to the hypertension induced by chronic inhibition of NOS with L-NAME [43]. Diosmetin decreased the blood pressure in the present study, which was associated with an improvement in vascular function in terms of endothelium-dependent vasorelaxation and sympathetic nerve-mediated vasoconstriction. These results are consistent with the finding that diosmetin has vasorelaxant effects in the porcine coronary artery [31]. In the present study, the vasodilator effects of diosmetin are relevant in reducing oxidative stress and raising endogenous antioxidant enzymes, subsequently increasing NO bioavailability in the rats that received L-NAME. The possible mechanism of oxidative stress presented in the L-NAME-treated rats might be associated with competitive binding at the L-arginine binding site caused an eNOS uncoupling, which stimulates O_2_^•−^ production in the vasculature. O_2_^•−^ quickly reacts with NO, forming ONOO-, and rapidly oxidizes tetrahydrobiopterin (BH4), which results in eNOS uncoupling. [44]. Moreover, it has been suggested that NO is not only a potent vasodilator, but that it also deactivates NE and suppresses sympathetic nerve overactivity in spontaneously hypertensive rats [45]. The antioxidant effects of diosmetin in the present study are consistent with the results of protein expression since the downregulation of Nrf2 and HO-1 protein expression in L-NAME rats was recovered in the diosmetin treated group. Nrf2 is a transcriptional factor that facilitates endogenous antioxidant enzymes [46] such as HO-1, SOD, and CAT. A major function of HO-1 is to metabolize heme-to-heme yields of iron, carbon monoxide, and biliverdin to mitigate oxidative stress [47]. HO-1 plays an important role in cell protection from oxidative stress. An increase in HO-1 expression associated with the reduction of reactive oxygen species has been reported in C2C12 myoblasts [48]. The depletion of HO-1 protein expression in the present study might be the consequence of Nrf2 expression downregulation. This could imply the imbalance of reactive oxygen species and antioxidant enzymes in this animal model. Moreover, it has been found that SOD and CAT activity are decreased in L-NAME-induced hypertension [49]. Moreover, several studies have reported that diosmetin has antioxidant properties relevant to the elevation of mRNA and the protein expression of Nrf2, HO-1, and NQO1 in human liver cells [50,51].

Vascular structural changes in L-NAME rats were observed, as shown by the thickness of the aortic wall and the accumulation of collagen in the aorta. This aortic remodeling was mitigated by treatment with diosmetin. From the results of this study, at two least possible mechanisms could support the effect of diosmetin on alleviating vascular remodeling. First, diosmetin reduced the blood pressure and subsequently decreased the pressure load on the vascular wall. Second, diosmetin reduced the oxidative stress and inflammation in L-NAME rats. Inflammation has been implicated in the structural remodeling process in L-NAME rats [52,53,54]. The relationship between oxidative stress and inflammation has been established as being that oxidative stress can induce inflammation [20]. From our results, excessive ROS could stimulate inflammation in the aorta and could mediate remodeling in L-NAME rats. Inflammation occurred in the aortic tissue, as supported by increasing aortic IL-6 in L-NAME rats. This was related to the upregulation of p-JNK/p-NF-κB protein expression in the aortic tissue. Diosmetin reduced inflammatory cytokines in the aortic tissue and downregulated the p-JNK/p-NF-κB protein expression. In fact, MAPK members such as extracellular regulated kinase1/2 (ERK1/2) and JNK are responsible for cellular proliferation and apoptosis [55]. NF-κB is a transcription protein that upregulates many genes associated with inflammation [56], such as IL-6. It has been reported that L-NAME hypertensive rats have elevated phosphor p-JNK and NF-κB protein expression, leading to tissue inflammation [20]. Our findings are supported by a previous study in which diosmetin prevented the expression of the NF-κB signaling pathway and suppressed the levels of inflammatory mediators and cytokines to alleviate hepatic histopathological changes in endotoxin-induced acute hepatic failure in mice [57]. From the results of this study, diosmetin at a dose 40 mg/kg was an effective dose or optimal dose since this dose produced a greater effect on reductions of blood pressure, vascular dysfunction, and oxidative stress than the lower dose (20 mg/kg) in L-NAME hypertensive rats. A 40 mg/kg dose of diosmetin in rats was calculated to 6.4 mg/kg as a concentration for beneficial effects at the food level in human [58].

Captopril, an ACEI, was used as a positive control agent for comparison with the effects of diosmetin. It reduced blood pressure and alleviated vascular dysfunction and remodeling in L-NAME rats. It is well established that captopril is an antihypertensive drug due to its chemical structure, which can inhibit ACE [6,59]. The cascular effects of captopril in the present study were consistent with previous studies [40,54]). Captopril also has indirect-vasorelaxant effects via increasing bradykinin production [60]. It has also demonstrated antioxidant and inflammatory effects that might be involved with restorations of Nrf2, HO-1, p-JNK, and p-NF-κB protein expressions in L-NAME rats. Additionally, the antioxidant properties of captopril have been supported by its sulfhydryl group [61].

The results of this study could indicate that diosmetin has similar effects to captopril in regard to lowering blood pressure, vascular changes, oxidative stress, and inflammation in hypertensive rats. Additionally, previous studies have reported that flavonoids and diosmetin exhibited an inhibitory effect on angiotensin converting enzyme activity [30,62].

## 5. Conclusions

**Figure 10:**
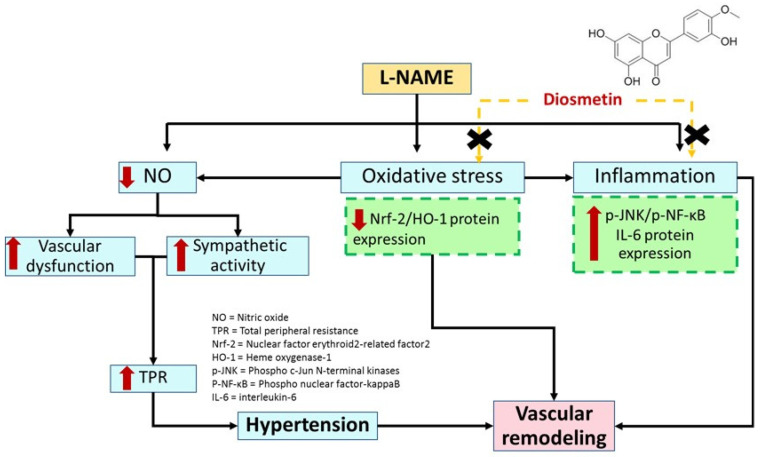
The possible mechanism action of diosmetin on vascular dysfunction and remodeling in L-NAME hypertensive rats.

In summary, diosmetin exhibited an antihypertensive effect in NO-deficient rats. It alleviated endothelium-dependent vasorelaxation and overactivity of sympathetic nerve-mediated vasoconstriction and aortic remodeling via reducing oxidative stress, raising NO bioavailability, and decreasing inflammation in L-NAME-treated rats. The possible molecular mechanisms were relevant in the restoration of Nrf2, HO-1, p-JNK, and p-NF-κB protein expression. The proposed mechanisms of diosmetin on blood pressure and vascular alteration were shown in Figure 10.

## Figures and Tables

**Table 1:** Hemodynamic parameters under anesthetization in all experimental groups.

Parameters	Control	LN + Vehicle	LN + Diosmetin20	LN + Diosmetin40	LN + Captopril
SP (mmHg)	120.57 ± 1.86	185.03 ± 3.83 ^a^	151.37 ± 3.49 ^ab^	144.22 ± 3.32 ^ab^	136.87 ± 2.44 ^abc^
DP (mmHg)	79.32 ± 1.96	125.91 ± 4.67 ^a^	109.73 ± 3.65 ^ab^	102.93 ± 5.16 ^ab^	95.19 ± 2.59 ^ab^
MAP (mmHg)	93.07 ± 1.72	145.61 ± 3.71 ^a^	123.61 ± 3.57 ^ab^	116.69 ± 2.71 ^ab^	109.08 ± 2.44 ^abc^
PP (mmHg)	41.21 ± 1.83	59.12 ± 5.06 ^a^	41.74 ± 0.98 ^b^	42.72 ± 1.26 ^b^	41.67 ± 1.08 ^b^
HR (beat/min)	344.32 ± 10.40	416.04 ± 16.34 ^a^	352.31 ± 12.70 ^b^	349.84 ± 5.99 ^b^	350.71 ± 7.97 ^b^

^a^ *p* < 0.05 vs. control group, ^b^ *p* < 0.05 vs. LN + vehicle group, ^c^ *p* < 0.05 vs. LN + diosmetin20 group. LN, L-NAME; LN + Diosmetin20, L-NAME treated with 20 mg/kg of diosmetin; LN + diosmetin40, L-NAME treated with 40 mg/kg of diosmetin; LN + captopril, L-NAME treated with captopril. (*n* = 8/group). Statistical analysis between the five groups was performed using the one-way ANOVA followed by Turkey’s post hoc test.

## Data Availability

Data are contained within the article.
